# Long-acting antipsychotic drugs for the treatment of schizophrenia: use in daily practice from naturalistic observations

**DOI:** 10.1186/1471-244X-12-122

**Published:** 2012-08-21

**Authors:** Giuseppe Rossi, Sonia Frediani, Roberta Rossi, Andrea Rossi

**Affiliations:** 1 U.O. Psichiatria, IRCCS “Centro San Giovanni di Dio” Fatebenefratelli, via Pilastroni 4, Brescia, Italy; 2 Centro di Salute Mentale La Badia U.S.L. 11, Empoli, Italy; 3 Medical Dept. Eli Lilly Italy, Via Gramsci 731, Sesto Fiorentino (FI), Italy

**Keywords:** Delayed-action preparations, Antipsychotic agents, Schizophrenia, Patients, Review

## Abstract

**Background:**

Current guidelines suggest specific criteria for oral or long-acting injectable antipsychotic drugs (LAIs). This review aims to describe the demographic and clinical characteristics of the ideal profile of the patient with schizophrenia treated with LAIs, through the analysis of nonrandomized studies.

**Methods:**

A systematic review of nonrandomized studies in English was performed attempting to analyze the factors related to the choice and use of LAIs in daily practice. The contents were outlined using the Cochrane methods for nonrandomized studies and the variables included demographic as well as clinical characteristics. The available literature did not allow any statistical analysis that could be used to identify the ideal profile of patients with schizophrenia to be treated with LAIs.

**Results:**

Eighty publications were selected and reviewed. Prevalence of LAI use ranged from 4.8% to 66%. The only demographic characteristics that were consistently assessed through retrieved studies were age (38.5 years in the 1970’s, 35.8 years in the 1980’s, 39.3 years in the 1990’s, to 39.5 years in the 2000’s) and gender (male > female).

Efficacy was assessed through the use of various symptom scales and other indirect measurements; safety was assessed through extrapyramidal symptoms and the use of anticholinergic drugs, but these data were inconsistent and impossible to pool. Efficacy and safety results reported in the different studies yielded a good therapeutic profile with a maximum of 74% decrease in hospital admissions and the prevalence of extrapyramidal symptoms with LAIs consistently increased at 6, 12, 18, and 24 months (35.4%, 37.1%, 36.9%, and 41.3%, respectively).

**Conclusions:**

This analysis of the available literature strongly suggests that further observational studies on patients with schizophrenia treated with LAIs are needed to systematically assess their demographic and clinical characteristics and the relationships between them and patient outcome.

Besides the good efficacy and safety profile of LAIs, health care staff must also take into account the importance of establishing a therapeutic alliance with the patient and his/her relatives when selecting the most appropriate treatment. LAIs seem to be a good choice not only because of their good safety and efficacy profile, but also because they improve compliance, a key factor to improving adherence and to establishing a therapeutic alliance between patients with schizophrenia, their relatives, and their health care providers.

## Background

Schizophrenia is a chronic disabling illness with a worldwide lifetime prevalence of about 1% [1]. It is characterized by relapses alternating with periods of partial or full remission [2] and poor adherence to antipsychotic treatment, leading to multiple rehospitalizations [3] and increased direct and indirect costs [4-11].

Antipsychotic medications have evolved in order to achieve a better control of schizophrenia. Long-acting injectable antipsychotic drugs (LAIs), introduced for the first time in the 1960’s, demonstrated their benefits by lowering relapse rates and durations of hospitalization [3,12-14], although the high prevalence and severity of extrapyramidal symptoms (EPS) made it necessary to develop new treatments. Second-generation antipsychotics (SGAs) provided a better tolerability profile compared to the first-generation antipsychotics (FGAs), but a high nonadherence rate was still observed [15]. About 57% of outpatients treated with oral SGAs adhere to the therapy after 6 months [16]. The efficacy and safety of LAIs have been well established through several randomized controlled trials and therapy choice criteria for these patients have been published recently [17], categorized by disease severity, patients’ socioeconomic level, and patients’ autonomy.

The recently released LAI SGAs provide the same favorable profile of oral SGAs, with an increased likelihood of improving antipsychotic treatment adherence [3,13].

To determine the ideal profile of the patient suitable for treatment with LAIs, several characteristics should be analyzed in order to understand which of these will predict a better clinical outcome. Relevant information about all these characteristics is available in the literature and important associations between these and LAIs have been established. These features include the following: compliance with and adherence to the treatment [18-21]; attitudes of psychiatrists [22-25], relatives [26], and/or patients [27,28] towards antipsychotic medication; patients’ insight about the disease [29-34]; history of relapses [35]; previous treatment with antipsychotic medication [36]; number of previous hospitalizations [3,37]; duration of illness [31]; presence of positive [35] and/or negative [38,39] symptoms; medication-related adverse events [31,40]; and patient experience with medication [41], among others.

Additionally, demographic features such as age, gender, ethnicity, educational background, insurance coverage, polypharmacy, and history of substance abuse should also be taken into account when considering treatment with LAIs [40,42-48].

The ideal candidate for LAI treatment should be a patient who has an unsatisfactory adherence to treatment and who needs to follow a treatment plan ensuring adequate control of the disease, fewer relapses, and good adherence to the treatment—factors that will translate into a better quality of life (QoL), fewer adverse events, fewer rehospitalizations, and a comprehensive improvement. To achieve this goal, a holistic approach has to be established with the aid of health care personnel, patient relatives, and the patients themselves.

The aim of this systematic review is to describe the profile of the ideal patient with schizophrenia to be treated with LAI through the naturalistic observation of age, gender, ethnicity, adherence to and compliance with LAI by both patient and physician and their relative perception of LAI together with treatment efficacy and safety profile.

## Methods

### Study design

A systematic literature review [49] was performed on the data reported in nonrandomized studies for the use of LAIs. The contents of this systematic review were outlined using Cochrane methods for nonrandomized studies [50] in line with the Meta-analysis of Observational Studies in Epidemiology (MOOSE) methods [51]. Nonrandomized studies were chosen because randomized clinical trials, having strict inclusion and exclusion criteria, are not likely to assess clinical judgment of the physicians in the routine clinical practice regarding patients suitable for LAI treatment.

### Search strategy

**Figure 1:**
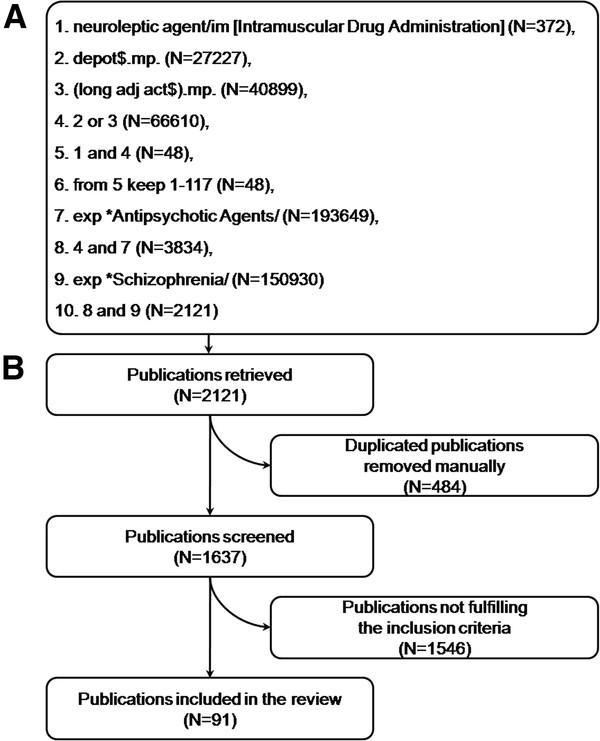
**Search strategy and results. A**. Search strategy and results in the OVID interface. **B**. Study selection and results algorithm.

A comprehensive literature search of the Medline (dating back to 1966), Embase (dating back to 1988), and Cochrane (dating back to 1964) databases was conducted using the OvidSP® interface (Ovid Technologies, Inc.; New York, NY) on May 15, 2011 by the medical information team at Primo Scientific Corporation (Panama City, Panama). The search strategy and results are summarized in Figure 1A. All the possible combinations of the key terms “neuroleptic agent” or “antipsychotic” and “depot” or “long-acting” or “injectable” or “extended release” were employed to obtain relevant reports, regardless of their publication type. The search was then restricted to nonrandomized studies published in English, reporting information on the use of LAIs for the different study variables fulfilling the inclusion criteria (ie, patient demographics; adherence to and compliance with LAI treatment; physician, patient, and relatives perception of LAIs; and the efficacy and safety profiles of LAIs). All the electronic or hard copy publications were retrieved by the library staff of Eli Lilly and Company (Indianapolis, IN) and no abstracts-only or personal communications with the authors were required.

### Inclusion and exclusion criteria

Inclusion criteria were nonrandomized studies (retrospective or prospective, observational, open-label, mirror-image, before and after, and/or case reports/series) in English about the use of LAIs in patients with schizophrenia, schizophreniform disorder, or schizoaffective disorder.

Studies were excluded if they were randomized or double-blind; investigated only pharmacokinetic or pharmacodynamic characteristics of LAIs; patients included were not treated by their psychiatrist according to usual clinical practice; and/or if patients had other psychiatric pathologies.

A flow chart is presented in Figure 1B to illustrate the study selection process and results. A detailed list of the studies included is presented in the Additional file 1. Abstracts and full-text articles were screened according to the inclusion and exclusion criteria and the publication full texts were retrieved for those matching the inclusion criteria. Various settings (eg, hospital-based LAI clinics, outpatient clinics) in different countries and populations (eg, young populations, patients in prison, pregnant women) were included in the nonrandomized studies, as these were not restricted by the inclusion criteria.

### Statistical analysis

A formal meta-analysis was not performed because only gender and age were consistently reported throughout the studies and these two characteristics have poor prognostic value to determine which patients benefit the most from the use of LAIs.

The available literature did not allow any statistical analysis that could be used to identify the ideal profile of patients with schizophrenia to be treated with LAIs. Only five of the publications retrieved (all publications derived from the European Schizophrenia Outpatient Health Outcomes [SOHO], a large observational study) included both patients using LAIs and patients using different oral antipsychotics [52-56]. This study did not report any statistical comparisons to assess differences in clinical or demographic characteristics between the two cohorts.

All of the studies retrieved were grouped per publication decade, and the weighted mean age of patients using LAIs was calculated for each decade to assess a possible trend.

### Limitations of the study

1) Study presents nonrandomized studies for the use of LAIs published in English

2) Literature available at resources Medline (dating back to 1966), Embase (dating back to 1988), and Cochrane (dating back to 1964) databases was used in this study

3) There are inconsistencies in reporting the drugs and dosages (in terms of absolute drug concentrations and mg of chlorpromazine equivalents [CPZE]) used in the different studies

4) Formal quality assessment of study could not be done as studies included are narrative in nature

5) Only gender and mean age are consistently reported among majority of the studies considered

6) Possible selection and information biases may exist as data are derived from nonsystematic, nonrandomized allocation to treatments and the existence of unobserved covariates that might have influenced the outcome

7) Some results were reported in only one paper and thus they must be interpreted/taken with caution

## Results

Eighty publications were included in this analysis (Additional file 1). Several studies assessed the prevalence of LAI use among patients with schizophrenia, which ranged from 11.9% to 66.0% [44,45,47,55,57-61]. Because of inconsistencies in reporting the drugs and dosages used in the different studies, it was not possible to perform comparisons regarding the doses of LAIs. As this study is narrative in nature [49], demographic and clinical characteristics were grouped without a formal assessment of study quality. All of the studies reporting either type of factors were included and discussed. With these findings in mind, identifying the characteristics of the ideal profile of the patient to receive treatment with LAIs entails analyzing several key demographic and clinical variables, trying to assess the main aspects of efficacy and safety of these drugs.

### Demographic aspects

Only gender and mean age were consistently reported in all of the studies, while other demographic factors were scarcely or inconsistently reported. The studies by Lindström et al., (1996) [57] and Sim et al., (2004) [58] report a higher proportion of male patients (58.15% [57]; 55.9% [58]) treated with LAIs as compared to females (41.85%; 44.1%) [57,58]. The calculated mean weighted age at treatment has been mostly stable throughout the decades, from 38.5 years in the 1970’s, 35.8 years in the 1980’s, 39.3 years in the 1990’s, to 39.5 years in the 2000’s. One article [62] reported that older age was associated with lower doses of LAIs (275 ± 239 mg CPZE) and, though no significant correlation of age with LAI daily dose for either haloperidol decanoate (28 days 3.5 ± 2.5 mg; p = .50) or fluphenazine decanoate (16 days 1.5 ± 1.3; p = .70) was found, a pooled analysis of LAIs revealed that patients using them in low doses (≤300 mg CPZE) were significantly older (p = .005) [44].

In terms of ethnicity, another study [47] reported that African American descent is a stronger predictor of FGA use (odds ratio [OR] 1.53; 95% confidence interval [CI] 1.12-2.09), at 53% higher doses (>1000 mg CPZE) than other ethnic groups [48], but less likely (p<.001) to receive LAIs or oral SGAs.

Results of a prospective comparative study of treatment for schizophrenia conducted from 1997 to 2003 in the use of first-generation depot antipsychotic at any time during the three-year study (N = 569) and those treated with only oral antipsychotics (N = 1,617) showed a low-education background (p<.001) and poor insurance coverage (p = .009) and further these were also reported to be significantly different among patients using LAIs versus oral antipsychotics (75% vs. 66% and 6% vs. 8%) [47]. Polypharmacy was also frequently reported [44] in patients with schizophrenia on LAI treatment and is a predictor for lower doses of antipsychotic drugs (OR 0.42; 95% CI 0.40-0.44; p<.001). Remington et al. [63] found differences in the setting where patients were treated: a large provincial hospital with 325 beds mostly devoted to treating patients with schizophrenia used significantly higher doses (p = .05) of antipsychotic drugs (N = 58; dose 773.8 ± 784.6 mg CPZE) when compared to a university-affiliated psychiatric teaching hospital with 85 beds (N = 52; dose 424.8 ± 317.2 mg CPZE), and to a 25-bed inpatient psychiatric unit (N = 53; dose: 355 ± 283.1 mg CPZE), respectively. Conventional and depot antipsychotic medication were the ones mostly used at provincial hospitals (99%; 43%), while the novel antipsychotics were the most commonly used at university and community hospitals (56%; 55%) [63]. Patients on LAIs were also found to receive higher doses of neuroleptic drugs in terms of chlorpromazine equivalent doses (525 mg CPZE; p<.001) [61].

### Clinical aspects

Several key clinical elements are reported as being associated with the use of antipsychotic treatment for schizophrenia.

#### Perception of LAIs

Less than 20% of patients with schizophrenia receive LAIs and less than 10% of psychiatrists offer LAIs after a first psychotic episode, prescribing them in a very conservative manner and only when other therapeutic options have failed [26], even though there is no evidence supporting this practice [64]. It is important to note that psychiatrists have decreased their LAI prescribing rates in the last five years by approximately 50%, although LAIs are shown to be very efficacious, are associated with higher compliance rates, and are currently more available [25]. A questionnaire-based survey of 198 German psychiatrists found that LAI was only prescribed to 13.3% out of 26.7% of patients with first episode schizophrenia [65]. Principal reasons for lower prescription rate were: difficulty in presenting the benefits of LAI to patients for first treatment option; poor availability of the LAI version of risperidone, olanzapine pamoate, and paliperidone palmitate [22,25,66], specifically preferred second generation LAI antipsychotics [AP2G] [67,68]; and lower preference of psychiatrists for prescribing LAI due to perception of adverse effects of depot antipsychotics and related impact on the relationship with the patient [65].

Psychiatrists prescribe oral tablet antipsychotics 88% of the time [26]. When LAIs are prescribed, SGAs are preferred (66%) over FGAs for LAI-naïve patients [25]. There is a report of patients having lower relapse rates after using LAI risperidone after 2 years of treatment following their first episode of psychosis [64].

Regarding patient attitudes toward LAI formulations, 24% of patients reported LAIs as their first choice of treatment, while 88% reported the acceptance of oral tablets as their first choice. There is a marked contrast when the patients are separated into three different groups (LAI-naïve patients, patients currently receiving LAIs, and patients previously treated with LAIs). LAI-naïve patients preferred oral tablets over LAIs (94% vs. 23%), whereas patients on LAI treatment chose it over oral tablets (73% vs. 67%). Compared to LAI-naïve patients, patients with previous LAI experience chose LAI more often, but curiously, had a greater preference for tablet formulations [36].

The results from a clinical survey evaluating the prescription rate of antipsychotics by psychiatrists corroborated the belief that patients with a poor course of schizophrenia fit the ideal profile to receive LAI treatment [69].

Patients with a better course of illness are characterized by having good insight about their illness, high educational levels, high levels of participation in treatment decisions, knowledge of their condition, and a desire to maintain a therapeutic alliance with the psychiatrist. A new approach could also include these patients as candidates to receive LAIs [45]. Many studies have reported that patients with better relationships with their physicians have better compliance rates compared to those with “fair” or “poor” relationships (74% vs. 26%, respectively) [21,70].

There is a high prevalence of poor insight among patients with schizophrenia [71]. Poor insight is the most common symptom of schizophrenia [34]; approximately 57% of patients are moderately or severely unaware of having a mental illness [33]. Poor insight has been related to poor compliance [72], which is the main cause of nonadherence to treatment in patients with schizophrenia [73]. The consequences of poor patient insight about their illness are reflected in a greater risk of relapse and more frequent and longer hospital admissions [21]. Treatment with LAI risperidone improved insight scores in patients previously treated with oral SGAs or typical LAIs. Additionally, insight improvement corresponded to favorable changes in other symptom domains, such as the Positive and Negative Symptoms Scale (PANSS) and the Clinical Global Impression (CGI)-Severity subscale [71].

#### Adherence to and compliance with LAI treatment

**Figure 2:**
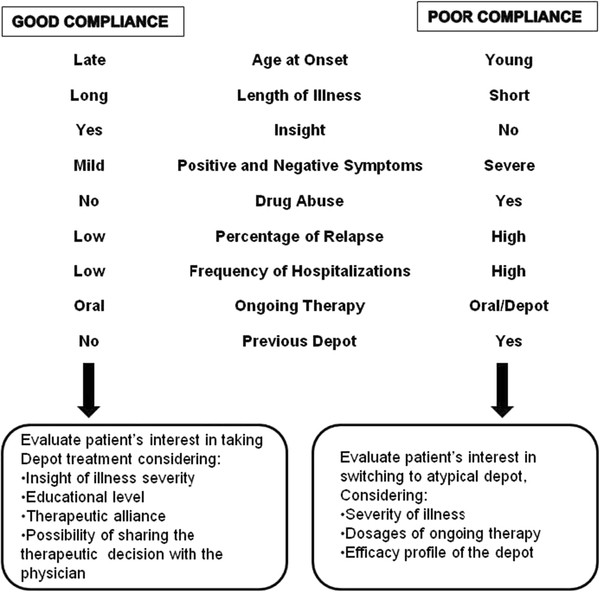
Decision algorithm for choosing a long-acting antipsychotic based on treatment compliance predictors reported in the literature.

Nonadherence rates with antipsychotics are often reported as being 40%-60% [74,75]. When comparing treatment adherence to preference between patients on oral antipsychotic tablets or LAIs, patients prescribed with LAIs were significantly more compliant than those receiving oral antipsychotics [11,27,40,76-78]. Figure 2 presents an algorithm of compliance predictors, that can help the mental-health specialists choose the best next step in the management of patients with schizophrenia on treatment, taking into consideration the most relevant aspects found in the literature. These predictors must be incorporated into the concept that all those involved in the integral care of the patient with schizophrenia must engage in a therapeutic alliance [21].

#### Efficacy

The efficacy of LAIs in general, and SGAs in particular, seems to be consistently shown throughout studies where patients reported improvement on the most common efficacy measures.

One such measure is the CGI scale, which has become one of the most widely used assessment tools in psychiatry since it was first published [79]. A greater baseline overall CGI score was found to be a predictor of discontinuation for antipsychotic medication [54]. Studies report that CGI scales significantly improved in patients using LAI SGAs [52,53,55,56,80], zuclopenthixol decanoate [81], perphenazine decanoate [82,83], or LAI risperidone [84,85]. PANSS, which is not an outcome rating instrument per se, but originated from the growing need to reduce the heterogeneity of what was known about schizophrenia, [86] was also used in observational studies [87]. PANSS score also consistently improved in studies where LAI risperidone was used [88,89]. The Comprehensive Psychiatric Rating Scale (CPRS) has also been used in another study [90], reporting significant correlations (p<.001) with the serum levels of zuclopenthixol decanoate.

Other authors have attempted to evaluate the QoL experienced by patients using LAIs. This has been assessed by different studies using various scales, such as the Global Assessment of Functioning (GAF), the Short Form Health Survey (SF-36), and the EuroQoL 5-Dimensions Visual Analog Scale (EQ-VAS). Studies on the use of LAIs in different settings have reported significant improvements (p<.01) in the total GAF score [47,79,91-94] or in the SF-36 overall score [95], as well as in some of its items [71,84,91,93,96]. Although the use of the EQ-VAS, another scale for assessing QoL used through the SOHO cohorts, has not yielded significant improvements in QoL for all pooled LAIs [52,53], its baseline value has been found to be a significant factor associated with achieving long-lasting symptomatic remission (OR 1.026; 95% CI not reported; p<.0001) [56].

An indirect measurement of efficacy is the hospitalization rate which, despite having different operational definitions, improved in patients treated with LAIs. Studies of treatment with LAIs consistently report significant decreases in the different quantifiers expressing the length of hospitalization (ie, absolute hospital days, days per patient, number of hospitalizations, bed-days per patient, or number of hospitalizations per patient) [9,47,80,85,97-106]. Only one small study (n = 46) found no statistical differences in rehospitalization rates between patients using oral antipsychotics versus LAIs [107]. A greater length of hospitalization was related to an increased number of prescriptions of LAIs versus oral antipsychotics and the history of psychiatric hospitalization in the prior year was a predictor of LAI FGA use [47]. The frequency of previous admissions was a predictor of rehospitalizations in a one-year observational trial [98]. The annual risks of readmission for patients using fluphenazine decanoate or haloperidol decanoate were 21% and 35%, respectively [46] and a decrease in rehospitalization rates was reported in studies with LAI fluphenazine (74% decrease in hospital admissions compared with previous treatment) [105], perphenazine (59% decrease in relative risk compared with haloperidol) [108]; and LAI risperidone (27% reduction in hospitalization rates) [9]. Patients using haloperidol decanoate presented statistically significantly greater differences in rehospitalization risk when compared to oral risperidone (Holm’s adjusted p = .001), olanzapine (Holm’s adjusted p = .0008), and clozapine (Holm’s adjusted p = .049) [46]. Frequency of hospitalizations was positively related with duration of illness, with current dose of neuroleptics (i.e., fluphenazine), and with overall neuroleptic exposure [109].

#### Safety

Many patients switching to LAI risperidone after treatment with conventional antipsychotics showed a significant improvement in EPS, measured primarily by the Extrapyramidal Symptom Rating Scale (ESRS) [90,91,93,95,96,110-113]. The ESRS has also been used in patients treated with perphenazine decanoate [82,83] and fluphenazine decanoate [114] with similar results. When the Drug-Induced EPS Scale (DIEPSS) was applied to patients treated with LAI risperidone, no significant differences were found in the rate of EPS [94]. In the SOHO study [53], the prevalence of EPS in the Italian cohort treated with LAIs consistently increased at 6, 12, 18, and 24 months (35.4%, 37.1%, 36.9%, and 41.3%, respectively). In the same cohort, the proportion of EPS at 12 months was significantly higher in patients treated with typical LAIs (32%) and oral typical antipsychotics (30%) compared to risperidone (16%), quetiapine (10%), clozapine (9%), and olanzapine (8%) [52]. Among patients with or without EPS treated with typical LAIs, the GAF and total BPRS scores differed at hospital discharge [115]. An indirect measure of EPS is the use of anticholinergic drugs, which was reported as significantly higher in patients using LAIs (47%) compared with patients using oral SGAs (13%) [116] and has a rate ranging from 30.9% to 32.0% for overall LAIs [52,117]. Lower values of anticholinergic drug use were reported in patients using LAI risperidone (5.2%) [118] and higher values were reported in those using fluphenazine decanoate (67.0%) [114]. Common EPS and non-EPS adverse events and their frequencies are summarized in Tables 1 and 2, respectively. The largest report on prolactin-related adverse events was found in the Italian cohort of the SOHO study (n = 3016) [52] after 12 months of treatment with different antipsychotic drugs (risperidone, quetiapine, clozapine, typical oral antipsychotic drugs, and typical LAI). A significant proportion of patients using typical LAIs reported prolactin-related adverse events, the most common being loss of libido, which was reported by 38.1% of these patients.

**Table 1:** Percentages of extrapyramidal adverse events reported for different long-acting antipsychotic drugs

**Drug**	**FL**	**FE**	**R**	**R**	**R**	**R y**	**R a**	**R**	**R**	**P**	**V**	**V**	**Z**
**Reference**	[131]	[132]	[80]	[132]	[90]	[91]	[91]	[107]	[133]	[77]	[8]	[58]	[76]
**n**	**16**	**5**	**19**	**87**	**249**	**66**	**351**	**725**	**529**	**42**	**53**	**368**	**19**
Dystonia	---	---	---	1.1	---	---	---	---	---	---	15.1	0.5	---
Akathisia	12.5	40.0	5.3	2.3	2.0	19.7	11.1	17.0	3.4	---	43.4	2.4	---
Parkinsonism/tremor	6.2	---	---	4.6	---	---	---	---	6.0	2.4	30.2	7.6	---
Tardive dyskinesia	---	---	---	3.4	---	---	---	---	---	---	---	3.0	15.8
Hypertonia	---	---	---	2.3	---	---	---	---	---	---	24.5	---	---

Abbreviations: *Z* = zuclopenthixol decanoate; *R* = risperidone; *y* = in young patients; *a* = in adult patients; *FL* = flupenthixol; *FE* = fluphenazine; *V* = various long-acting antipsychotic drugs; --- = not reported.

**Table 2:** Percentages of the most frequent and selected general adverse events reported for different long-acting antipsychotic drugs

**Drug**	**FL**	**R**	**R**	**R**	**R, in no r**	**R in r**	**R**	**R vs oral**	**R**	**R**	**R y**	**R a**	**R**	**R**	**R**	**R**	**V**	**Z**
**Reference**	[131]	[15]	[79]	[86]	[84]	[84]	[85]	[88]	[89]	[90]	[91]	[91]	[105]	[107]	[132]	[133]	[88]	[76]
**n**	**16**	**715**	**192**	**202**	**312**	**82**	**1476**	**100**	**40**	**249**	**66**	**351**	**336**	**725**	**87**	**529**	**565**	**19**
Anxiety	12.5	12	12	15	28.7	24.5	6.9	11.0	---	11	21	23	22.3	26	16.1	23.9	---	---
Insomnia	6.2	10	9	9	26.5	25.0	7.0	9.0	5.0	6	29	19	23.5	22	16.1	18.7	---	8
Disease exacerbation	---	---	10	---	20.1	15.8	6.1	5.0	---	6	12	12	18.2	15	19.5	7.4	---	---
Depressive reactions	17	---	6	---	19.8	15.7	---	5.0	---	---	17	14	19.3	15	---	11.3	---	25
Headache	---	---	---	---	14.7	14.7	---	---	2.5	6	21	11		---	11.5	7.7	---	---
GI symptoms^a^	---	---	---	---	---	---	---	---	---	---	11	3	---	---	---	---	---	17
Glucose-related AEs	---	---	---	0.5^b^	---	---	---	---	---	---	---	---	---	---	0.0	0.8^c^	0.0	---
Prolactin-related AEs^d^	---	---	---	0.5	---	---	---	6.0	2.5^f^	2.8	---	---	---	---	---	---	0.4^e^	---
Sedation/Somnolence	---	---	---	---	---	---	---	---	---	---	12	5	---	---	---	---	---	---
Rhinitis	---	---	---	---	---	---	---	---	---	---	18	11	---	---	---	---	---	---
Fatigue	---	---	---	---	---	---	---	---	---	---	15	7	---	---	---	---	---	---
Others	6.2^h^	---	---	---	---	---	---	---	2.5^g,h^	---	---	---	---	---	---	---	---	17^h^

Abbreviations: *FL* = flupenthixol; *R* = risperidone; *r* = remission; *y* = in young patients; *a* = in adult patients; *V* = various; *Z* = zuclopenthixol decanoate; *GI* = gastrointestinal; *AEs* = adverse events; --- = not reported.

^a^Includes dyspepsia, nausea, and vomiting.

^b^New onset type 2 diabetes mellitus (n = 1).

^c^New onset type 2 diabetes mellitus (n = 3) and hyperglycemia (n = 1).

^d^Includes loss of libido, sexual impotence, galactorrhea, and gynecomastia.

^e^Impotence (n = 1) and galactorrhea (n = 1).

^f^Irregular menstruation (n = 1).

^g^Disturbances in accommodation.

^h^Dizziness.

For LAIs, small but statistically significant increases in body weight and BMI have been reported; contrasting reports of nonsignificant increases in these parameters were also found in the literature (Table 3). These weight and BMI increases were not dose-dependent [117].

**Table 3:** Weight and BMI changes reported in selected LAI publications

**LAI(s) used**	**Reference**	**n**	**Weight change, mean difference, kg**	**BMI change, mean difference, kg/m**^**2**^
Risperidone	[15]	715	1.4^***^	0.5^***^
Various	[48]	293	0.0^a^	NR
Various	[52]	176	0.1	NR
Various	[53]	540	2.9^b^	0.7^b^
Various	[55]	1173	NR	0.2^b^
Various	[58]	2399	−1.0^b^	NR
Various	[61]	534	1.3^c^	NR
Perphenazine	[78]	42	No weight change^d^	NR
Risperidone	[79]	192	0.0	−0.1
Risperidone	[85]	1476	0.9	0.3
Risperidone	[86]	202	0.5	0.2
Various	[88]	565	0.9^*^	NR
Risperidone	[90]	249	1.4^***^	0.5^***^
Risperidone	[105]	336	2.5	0.8
Risperidone	[108]	50	NR	0.0
Various	[112]	97	NR	1.5^b^
Risperidone	[132]	67	−0.3	NR
Risperidone	[133]	529	1.0^***^	0.6^***^
Various	[134]	5950	NR	−0.7^e^

Abbreviations: *BMI* = body mass index; *LAI* = long-acting injectable antipsychotic drugs; *NR* = not reported; *pts* = patients.

^a^Pts treated with in-range chlorpromazine-equivalent doses versus patients treated with higher chlorpromazine-equivalent doses.

^b^Versus oral antipsychotics.

^c^Pts treated with LAI and anticholinergic drugs versus patients treated with LAI without anticholinergic drugs.

^d^This study reported no weight change, with 16 pts gaining a range of 1-9 kg and 7 pts losing a range of 1-2 kg.

^e^Patients achieving persistent remission versus patients with prolonged disease.

^*^p ≤ .05; ^**^ p ≤ .01; ^***^p ≤ .001.

Pain caused by injections is another safety measure reported that is most often evaluated through the visual analog scale (VAS). One study [119] found that zuclopenthixol injections were significantly more painful than flupenthixol, fluphenazine, and haloperidol and that pain score measured by VAS was correlated to the Hamilton Scale for Depression (HAM-D). Another study found that injection-site pain with LAI risperidone significantly decreased over 50 weeks [96]. An additional LAI pooled study (haloperidol, fluphenazine, zuclopenthixol, and flupenthixol) found no significant correlation between VAS measures 5 minutes after the injection, but found a significant correlation after 2 days of the injection administration [119]. No relationship was found between VAS pain score, patient weight, or the score in the Udvalg for Kliniske Undersøgelser (UKU) 48-Item Side-Effect Rating Scale [8].

Some studies report the results while using LAIs in peculiar situations. One report [120] on the use of LAI risperidone during pregnancy was found, and it did not report any immediate complication at birth. Another study [121] found that mean serum copper levels were significantly (p = .004) higher in patients treated with fluphenazine decanoate (117.4 μg/dL; 95% CI 111.2-123.6 μg/dL) versus healthy controls (105.6 μg/dL; 95% CI 96.9-114.2 μg/dL); mean serum zinc levels did not differ between the two groups.

## Discussion

Long-acting injectable antipsychotic drugs were developed to become a therapeutic option especially for those patients where nonadherence is a major clinical concern, with consequences of subsequent relapse episodes and rehospitalizations [46,122,123]. Although there is a strong, but perhaps misguided, belief that LAIs cannot be used as first-line treatment for schizophrenia, new guidelines state that the clinician should consider offering them to LAI-naïve patients with schizophrenia [124-131].

The most favorable conditions for the development of an effective therapeutic alliance occur in patients who are well-informed about their illness and are compliant with treatment, in conjunction with medical staff who are willing to provide explanations and to offer a holistic therapeutic approach [132-134]. This type of therapeutic alliance promotes the patient’s adherence to their treatment and facilitates open lines of communication, so that patients can meaningfully participate in treatment decisions.

Even though nonrandomized studies have some well-known limitations (uncertainty of data collected, differences in their design and implementation, lower level of evidence than randomized controlled studies, and not being included in study registries) [50], they are the only studies in which patients were enrolled and treated according to physicians’ clinical judgment. Consequently, nonrandomized studies are the only studies that, in a naturalistic setting, allow researchers to understand how physicians, based on their clinical judgment, select patients with schizophrenia who have the ideal profile for LAI treatment and who should benefit the most from it. Moreover, they provide naturalistic observation of efficacy, safety and compliance to the treatment chosen by the physician as the most appropriate for each patient.

We found no obvious explanation for the decrease in the prescription rates of LAIs by psychiatrists [25,57,135] even when LAIs have specific advantages over oral FGAs and SGAs, especially in noncompliant patients [136,137]. This could also be due to the perception that depots and LAIs are old-fashioned and they are reserved for the most chronic and disabled patients [138-140]. Furthermore, due to higher availability of oral atypical antipsychotics in the 1990s, with associated heavy marketing, the decline in prescription of depot has continued due to the assumptions that patients always prefer oral to depots even though clinician’s attitude towards these medications is marginally improved. The adverse impacts of patient preference of depots to LAIs, in relation to relapse prevention are rarely explained and discussed with the patient [141]. A six-month, open-label study with LAI risperidone in 382 patients with recent schizophrenia or schizoaffective disorder (diagnosed ≤3 years ago) showed significant decrease of severity of schizophrenic symptomatology in 73%: among these, in 40% of patients, total PANSS score decreased by at least 20%. At the same time, patients showed an improvement of overall functioning, quality of life, and satisfaction [142,143]. These observations suggest that there is a need for prescribing LAI for effecting an increase in efficacy and functioning.

Even when the validity of using certain scales as efficacy endpoint measurements has been criticized [86,87], these and other efficacy endpoints such as good symptomatic control, improved QoL, and reduced hospitalization rates seem to be consistently achieved by patients with schizophrenia treated with LAIs.

Safety, which can be evaluated using different tools, might be a concern when considering the use of LAIs. The frequency of EPS seems to be reduced when patients switch from oral to LAIs and the most frequent EPS reported were akathisia and parkinsonism/tremor (Table 1). Tardive dyskinesia was generally reported less frequently, but was more commonly reported in patients using zuclopenthixol decanoate than those using LAI risperidone or LAIs in general [81]. Other frequently reported side effects of LAIs are anxiety, insomnia, disease exacerbation, depression, headache, and weight gain, but the data retrieved cannot be considered conclusive. It is fundamental to consider side effects, since patient attitude towards these strongly influence their attitude towards the entire treatment [8].

Unfortunately, the data reported in the literature cannot be combined any further to identify the ideal profile of the patient using LAIs, due to the inconsistency in the way the results are reported. Even those variables, such as age and gender, reported in all studies retrieved have poor prognostic value in terms of understanding how physicians select the ideal profile of the patient with schizophrenia to be treated with LAIs. The only consistently reported reason why certain patient profiles are identified as “ideal” to receive LAI treatment is because this type of administration ensures treatment delivery. Patient insight, attitude toward, and compliance with LAIs are crucial factors that influence physician’s LAI choice, especially when a good therapeutic alliance has been established [69,144]. Nowadays, the therapeutic alliance seems to be the most influential factor related to successful compliance and adequate adherence to the schizophrenia treatment [16,26,28,43,69]. This factor can improve efficacy, safety, and cost-effectiveness outcomes (Figure 2) [5-7,9,69,122].

**Figure 3:**
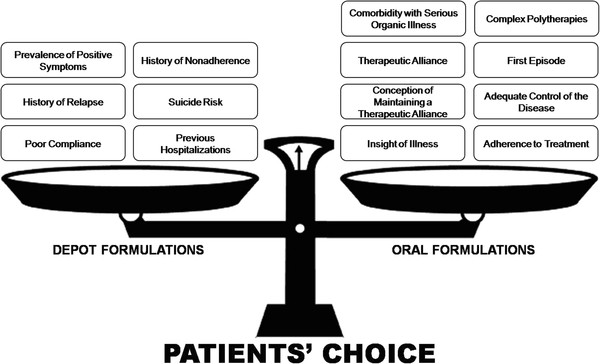
**Elements in the selection of long-acting injectable or oral formulations.** Necessary elements to be explained by mental health staff to patients and relatives before deciding the next best step in a drug treatment must be carefully weighted by the mental health staff, taking into account the patient’s choice.

Authors agree that the most common reason for prescribing LAIs is noncompliance, but these drugs on their own do not overcome the problem of treatment nonadherence. This is why therapeutic alliance plays a key role [16,26,28,43,69]. Greater illness insight is related to better attitudes toward treatment, improved compliance, and fewer relapses and hospitalizations [29,30]. Patients’ and their relatives’ opinions must be taken into consideration before initiating or switching to a new therapeutic plan. A survey among patients with schizophrenia revealed that 67% of them had not received any information about LAI treatment from their psychiatrists [25]. Ultimately, patients have the right to make an informed decision about LAI treatment. Its overall negative perception could be reduced if psychiatrists were to provide broader and more comprehensive information to patients with schizophrenia and their relatives. Furthermore, patients’ decisions regarding treatment should be guided by healthcare providers (Figure 3), after the most relevant topics and concerns have been evaluated, discussed and understood by the patients and their relatives.

## Conclusions

Though LAI prescription rates have been decreasing, they still prove to have good efficacy and safety profiles. The important question, therefore, is to determine which patients with schizophrenia are the most suitable for treatment with LAIs. Our research might reveals indicators that could support psychiatrists tailor their answer to this question when dealing with particular cases. Psychiatrists seeking to improve the therapeutic alliance with patients who need to improve treatment compliance and adherence should consider LAIs when establishing a therapeutic plan. Efficacy and safety must also be taken into account. Naturalistic setting trials aimed at understanding how therapeutic alliance can be improved by the use of LAIs should be carried out in order to systematically identify those patients who would most benefit from the use of LAIs.

## Competing interests

This article was sponsored by Eli Lilly and Company.

Giuseppe Rossi and Sonia Frediani have acted as invited speakers for Eli Lilly.

Andrea Rossi is a full time employee at Eli Lilly Italy.

Roberta Rossi declares no conflicts of interest.

## Authors’ contributions

GR designed the article structure, substantially revised it and provided all clinical guidance’s from retrieved literature.SF and RR contributed to the study design, literature revision and selection, and provided clinical decision guidelines. AR performed literature search, contributed in writing the article, revised and selected retrieved literature. All authors approved the final version of this article.

## Pre-publication history

The pre-publication history for this paper can be accessed here:

http://www.biomedcentral.com/1471-244X/12/122/prepub

## Supplementary Material

Additional file 1 **Observational studies included in the data analysis.** This data table includes the 80 studies that fulfilled the inclusion criteria for this review and their general description. Click here for file
