# Supplementary material for: Long-acting antipsychotic drugs for the treatment of schizophrenia: use in daily practice from naturalistic observations
Source: BMC Psychiatry. 2012 Aug 21;12:122. doi: 10.1186/1471-244X-12-122 (PMC3573926; doi:10.1186/1471-244X-12-122)
Supplement: Additional file 1 — Observational studies included in the data analysis. This data table includes the 80 studies that fulfilled the inclusion criteria for this review and their general description. [file 1471-244X-12-122-S1.doc]

**Table 1 – Percentages of extrapyramidal adverse events reported for different long-acting antipsychotic drugs**

| **Drug** | **FL** | **FE** | **R** | **R** | **R** | **R y** | **R a** | **R** | **R** | **P** | **V** | **V** | **Z** |
| --- | --- | --- | --- | --- | --- | --- | --- | --- | --- | --- | --- | --- | --- |
| **Reference** | **[131]** | **[132]** | **[80]** | **[132]** | **[90]** | **[91]** | **[91]** | **[107]** | **[133]** | **[77]** | **[8]** | **[58]** | **[76]** |
| **n** | **16** | **5** | **19** | **87** | **249** | **66** | **351** | **725** | **529** | **42** | **53** | **368** | **19** |
| Dystonia | --- | --- | --- | 1.1 | --- | --- | --- | --- | --- | --- | 15.1 | 0.5 | --- |
| Akathisia | 12.5 | 40.0 | 5.3 | 2.3 | 2.0 | 19.7 | 11.1 | 17.0 | 3.4 | --- | 43.4 | 2.4 | --- |
| Parkinsonism/tremor | 6.2 | --- | --- | 4.6 | --- | --- | --- | --- | 6.0 | 2.4 | 30.2 | 7.6 | --- |
| Tardive dyskinesia | --- | --- | --- | 3.4 | --- | --- | --- | --- | --- | --- | --- | 3.0 | 15.8 |
| Hypertonia | --- | --- | --- | 2.3 | --- | --- | --- | --- | --- | --- | 24.5 | --- | --- |

Abbreviations: Z = zuclopenthixol decanoate; R = risperidone; y = in young patients; a = in adult patients; FL = flupenthixol; FE = fluphenazine; V = various long-acting antipsychotic drugs; --- = not reported.

**Table 2 – Percentages of the most frequent and selected general adverse events reported for different long-acting antipsychotic drugs**

| **Drug** | **FL** | **R** | **R** | **R** | **R, in no r** | **R in r** | **R** | **R vs oral** | **R** | **R** | **R y** | **R a** | **R** | **R** | **R** | **R** | **V** | **Z** |
| --- | --- | --- | --- | --- | --- | --- | --- | --- | --- | --- | --- | --- | --- | --- | --- | --- | --- | --- |
| **Reference** | **[131]** | **[15]** | **[79]** | **[86]** | **[84]** | **[84]** | **[85]** | **[88]** | **[89]** | **[90]** | **[91]** | **[91]** | **[105]** | **[107]** | **[132]** | **[133]** | **[88]** | **[76]** |
| **n** | **16** | **715** | **192** | **202** | **312** | **82** | **1476** | **100** | **40** | **249** | **66** | **351** | **336** | **725** | **87** | **529** | **565** | **19** |
| Anxiety | 12.5 | 12 | 12 | 15 | 28.7 | 24.5 | 6.9 | 11.0 | --- | 11 | 21 | 23 | 22.3 | 26 | 16.1 | 23.9 | --- | --- |
| Insomnia | 6.2 | 10 | 9 | 9 | 26.5 | 25.0 | 7.0 | 9.0 | 5.0 | 6 | 29 | 19 | 23.5 | 22 | 16.1 | 18.7 | --- | 8 |
| Disease exacerbation | --- | --- | 10 | --- | 20.1 | 15.8 | 6.1 | 5.0 | --- | 6 | 12 | 12 | 18.2 | 15 | 19.5 | 7.4 | --- | --- |
| Depressive reactions | 17 | --- | 6 | --- | 19.8 | 15.7 | --- | 5.0 | --- | --- | 17 | 14 | 19.3 | 15 | --- | 11.3 | --- | 25 |
| Headache | --- | --- | --- | --- | 14.7 | 14.7 | --- | --- | 2.5 | 6 | 21 | 11 |  | --- | 11.5 | 7.7 | --- | --- |
| GI symptoms^a^ | --- | --- | --- | --- | --- | --- | --- | --- | --- | --- | 11 | 3 | --- | --- | --- | --- | --- | 17 |
| Glucose-related AEs | --- | --- | --- | 0.5^b^ | --- | --- | --- | --- | --- | --- | --- | --- | --- | --- | 0.0 | 0.8^c^ | 0.0 | --- |
| Prolactin-related AEs^d^ | --- | --- | --- | 0.5 | --- | --- | --- | 6.0 | 2.5^f^ | 2.8 | --- | --- | --- | --- | --- | --- | 0.4^e^ | --- |
| Sedation / Somnolence | --- | --- | --- | --- | --- | --- | --- | --- | --- | --- | 12 | 5 | --- | --- | --- | --- | --- | --- |
| Rhinitis | --- | --- | --- | --- | --- | --- | --- | --- | --- | --- | 18 | 11 | --- | --- | --- | --- | --- | --- |
| Fatigue | --- | --- | --- | --- | --- | --- | --- | --- | --- | --- | 15 | 7 | --- | --- | --- | --- | --- | --- |
| Others | 6.2^h^ | --- | --- | --- | --- | --- | --- | --- | 2.5^g,h^ | --- | --- | --- | --- | --- | --- | --- | --- | 17^h^ |

Abbreviations: FL = flupenthixol; R = risperidone; r = remission; y = in young patients; a = in adult patients; V = various; Z = zuclopenthixol decanoate; GI = gastrointestinal; AEs = adverse events; --- = not reported.

^a^Includes dyspepsia, nausea, and vomiting.

^b^New onset type 2 diabetes mellitus (n=1).

^c^New onset type 2 diabetes mellitus (n=3) and hyperglycemia (n=1).

^d^Includes loss of libido, sexual impotence, galactorrhea, and gynecomastia.

^e^Impotence (n=1) and galactorrhea (n=1).

^f^Irregular menstruation (n=1).

^g^Disturbances in accommodation.

^h^Dizziness.
